# Accelerometer Measured Sedentary and Physical Activity Behaviors of Working Patients after Total Knee Arthroplasty, and their Compensation Between Occupational and Leisure Time

**DOI:** 10.1007/s10926-020-09924-9

**Published:** 2020-09-18

**Authors:** T. H. Hylkema, S. Brouwer, C. M. Kooijman, A. J. De Vries, F. Breukelman, H. Dekker, J. Almansa, P. P. F. M. Kuijer, S. K. Bulstra, M. Stevens

**Affiliations:** 1 Department of Orthopedics, University Medical Center Groningen, University of Groningen, PO Box 30.001, 9700 RB Groningen, The Netherlands; 2 Department of Community and Occupational Medicine, University Medical Center Groningen, University of Groningen, Groningen, The Netherlands; 3 Department of Orthopedics, Nij Smellinghe Hospital, Drachten, The Netherlands; 4 Department of Orthopedics, Martini Hospital Groningen, Groningen, The Netherlands; 5 Department of Orthopedics, Wilhelmina Hospital Assen, Assen, The Netherlands; 6 Department of Orthopedics, Ommelander Ziekenhuishuisgroep Delfzijl, Delfzijl, The Netherlands; 7 Coronel Institute of Occupational Health, Amsterdam Public Health Research Institute, Amsterdam UMC, University of Amsterdam, Amsterdam, The Netherlands

**Keywords:** (*MeSH*): knee replacement, Accelerometry, Physical activity, Sedentary behavior

## Abstract

*Purpose* Objective measurements of sedentary and physical activity (PA) behavior are scarce among working-age patients who undergo total knee arthroplasty (TKA). Aim was to assess sedentary and PA behaviors using accelerometers and to identify compensation effects between occupational and leisure time of sedentary and PA behavior.* Methods* One year post-TKA, 51 patients wore an ActiGraph(GT3x) accelerometer for 7 days. Sedentary time, prolonged sedentary bouts (≥ 30 min) and PA (light-intensity and moderate-to-vigorous PA) were examined. Compliance with the guideline of > 150 min moderate-to-vigorous PA per week was calculated. Compensation effects were analyzed using multilevel models, splitting effects into routine and within-day compensation, stratifying by physical and non-physical jobs. The routine compensation effects are the ones of interest, representing habitual compensation during a week. *Results* Participants spent 60% of time in sedentary bouts and 17% in prolonged sedentary bouts, with 37% of PA spent in light-intensity and 3% in moderate-to-vigorous activity. About 70% of patients met the PA guideline. Routine compensation effects were found for workers in physical jobs, who compensated for their occupational light-intensity PA with less light-intensity PA during leisure time. Workers in non-physical jobs did not compensate for their occupational prolonged sedentary bouts, as these continued during leisure time. * Conclusion* This study showed that working TKA patients are highly sedentary 1 year after surgery, but most met the PA guideline. Especially those with non-physical jobs do not compensate for their occupational prolonged sedentary bouts. This stresses the need to stimulate PA among TKA patients not complying with the guidelines and those with non-physical jobs.

## Introduction

Total knee arthroplasty (TKA) procedures for end-stage knee osteoarthritis are among the most common elective surgical procedures in Western societies. For most patients TKA is highly successful in restoring quality of life by relieving pain and improving function, but it is also known that approximately 20% do not achieve good functional outcomes [1]. To date, working-age patients represent the fastest-growing group of TKA recipients. This trend is set to continue, with predictions that over half of TKAs in the United States will be performed on patients younger than 65 [2, 3]. Similar trends are observed and expected elsewhere in the West [4, 5]. Beyond pain relief and functional improvements, increasing physical activity (PA) levels is considered to be an important goal after surgery [6, 7]. Regular PA whereby health-enhancing guidelines for PA are reached, is associated with substantial health benefits such as increased functional mobility, decreased pain and lower rates of depression [8]. Specifically for TKA patients, PA benefits bone quality and implant fixation [9].

Two studies, using self-reported questionnaires such as the University of California Los Angeles (UCLA) activity score, examined PA levels among working-age TKA patients in Canada (< 69 years) and the United Kingdom (< 65 years) [7, 10]. The disadvantage of questionnaires assessing PA is that most of them do not examine any information on occupational physical activity or sedentary behavior, nor provide insight into PA intensities. Moreover, they are prone to recall and social desirability bias, which results in overestimation of PA [11, 12]. To gain deeper insight into postoperative sedentary behavior and PA intensities, accelerometers are viewed as the most reliable objective measure of PA and prevent self-reported overestimation [13, 14]. These small activity monitors can measure bouts of prolonged sedentary time (≥ 30 min), light-to-vigorous and moderate-to-vigorous PA. To date, assessment of PA using accelerometers has not been performed among working-age TKA recipients.

In addition to measuring PA levels objectively, it is also particularly interesting to focus on both occupational time and leisure-time PA among working TKA patients. Occupational PA accounts for a major part of total PA energy expenditure, as full-time employees spend almost half of their waking time at work [15]. This implies that, as work has become increasingly sedentary, one-third to half of our daily sitting occurs at work [16]. Prolonged periods of sedentary behavior may lead to functional decline and compound disability through deconditioning [17, 18]. To find the right balance between occupational and leisure-time PA and to prevent an inactive lifestyle, it is commonly believed that there are compensation effects between the two domains of PA [19]. This also implies that when workers engage in a large amount of occupational PA, they will be less active or even mostly sedentary during leisure time, and vice versa. Although several studies have focused on this topic, findings are still inconsistent and probably not specifically applicable to TKA patients [20, 21]. Hence the association between occupational and leisure time with respect to sedentary and PA behavior remains unclear.

Increased understanding of sedentary and PA behavior of working-age TKA patients, including compensation effects between occupational and leisure time, will help inform health professionals to support and promote healthy PA behavior among working TKA patients. This study therefore aimed to: 1. assess sedentary and PA behavior using accelerometer measurements among working-age TKA patients for a week, dividing it into occupational and leisure time, and 2. identify any compensation effects between occupational and leisure-time PA.

## Materials and Methods

### Study Design and Participants

An observational repeated-measures study was performed in the northern Netherlands between 2017 and 2018. Patients were recruited from four hospitals: Martini Hospital Groningen (large teaching hospital), Ommelander Ziekenhuis Groep Winschoten/Delfzijl (general hospital), Nij Smellinghe Hospital Drachten (general hospital) and Wilhelmina Ziekenhuis Assen (general hospital). Inclusion criteria were being between ages 18 and 65, having undergone TKA surgery approximately in the last 12 months, having returned to work after surgery, and working a minimum of 24 contractual hours per week. Exclusion criteria were total hip arthroplasty or contralateral TKA in the last 6 months, being more than 3 months pregnant, partial sick leave, or an occupation where an accelerometer could not be worn.

### Procedure

Patients were asked to participate in the study by responding to a formal invitation letter, with addresses derived from medical records. All letters were sent between 6 and 12 months postoperatively. Patients willing to participate were called by the first researcher (TH) to plan a visit. All patients were visited at home or at work by the same researcher (TH) around 12 months postoperatively to bring the accelerometer and give verbal instructions on how to wear it, and hand out a questionnaire and log on occupational and leisure time. After the wearing period of 14 days, accelerometers were picked up at home or at work by one of the researchers.

### Ethics, Consent and Permissions

The study was approved by the Medical Ethical Committee of University Medical Center Groningen (METc 2014/182) and in accordance with the 1964 Helsinki declaration and its later amendments or comparable ethical standards. Informed consent was obtained from all patients at the first home or work visit.

### Measurements

#### Activity Monitoring

Physical activity was objectively measured using GT3x ActiGraph (Pensacola, FL) triaxial accelerometers. Participants were asked to wear the accelerometer on their contralateral hip at all waking hours except when bathing or engaging in water activities. We chose the contralateral hip as we assumed that wearing the accelerometer on the TKA side might influence the results if patients limp with the affected leg. They wore the device for 14 consecutive days to assess PA behaviors during and outside work time. They were also asked to fill out a log indicating the time they wore the device each day along with their work schedule and exercise activity. If logs were filled in wrongly or incompletely, work schedules were corrected based on a normal work schedule the participant verbally reported, judged by two authors (e.g. Monday–Friday 8 AM to 5 PM). Participants with at least 7 valid days (5 weekdays and 2 weekend days) including a minimum of 10 h wearing time per day were included in the analysis [22]. Non-wearing bouts were classified as periods of 90 consecutive minutes of zero counts per minute (cpm), allowing for up to 2 min of < 100 cpm [23]. Validated accelerometer thresholds were used to define sedentary as < 100 cpm, light-intensity PA as 100–1951 cpm, moderate-intensity PA as 1952–5724 cpm and vigorous-intensity PA as ≥ 5724 cpm [24]. Moderate and vigorous PA were merged into moderate-to-vigorous PA (MVPA). Prolonged bouts of sedentary time were defined as uninterrupted periods of < 100 counts lasting 30 min or more [25].

#### Covariates

Participants were asked to fill in a questionnaire including items on age, gender, marital status (married, cohabiting with partner or partner and children, or no relationship), having children (yes or no), educational level (categorized into low, i.e. primary school and lower vocational education; medium, i.e. secondary vocational education; and high, i.e. higher vocational education and university), BMI, comorbidity and job type. Body weight and body height, used to calculate body mass index (BMI) [categorized into normal (18.5–24.9), overweight (25.0–29.9) and obese (> 30.0)], and comorbidity were measured using a 28-item chronic conditions questionnaire [26]. Comorbidity was categorized as no, one or two, and more than two comorbidities. Job type included the job description and was classified into physical and non-physical jobs. This classification was conducted blinded by two authors (TH and PK), based on job description. Examples of physical jobs are: nurse, farmer, order-picker, lorry driver, carpenter. Examples of non-physical jobs are: psychologist, secretary/clerk, IT-specialist, planner, owner insurance company. The overall agreement was 93% (53/57). Disagreement existed in four cases, with further explanation on job tasks given by one researcher (TH) who visited all participants at work. Consensus could be established thereafter.

### Statistical Analysis

Descriptive statistics were used to summarize the sociodemographic and work-related characteristics of the participants with means and standard deviations (SD) or percentages.

To answer the first research question—the amounts of time spent in sedentary behavior—prolonged sedentary bouts ≥ 30 min, light-intensity PA or MVPA were presented descriptively as [mean (SD)] of the proportion of the wearing time. Means were presented for total wearing time (Monday–Sunday) and stratified by occupational time (Monday–Friday) and leisure time (Monday–Sunday). Leisure time included everything outside occupational times as reported in the patients’ log, including commuting. No occupational PA levels were reported for weekend days, while almost nobody worked during the weekend. To determine whether a participant met the minimum levels of PA recommended to maintain good health, the updated American Physical Activity Guideline (2018) was used. This guideline states that patients need to perform at least 150 min of MVPA for a minimum of 5 days/week [8]. This guideline was chosen because it recommends PA for adults and is internationally accepted, also in the Netherlands.

To answer the second research question—the compensation between occupational and leisure time with respect to sedentary and PA behavior—the days (Monday–Friday) in which individuals had both occupational and leisure time during the same day were selected. As outcome variable we used the proportion of wearing time over the entire day, modeling the course of sedentariness and PA during occupational and leisure times in a parallel growth model [27]. In this model both sedentary and PA levels (occupational and leisure time) are modelled longitudinally over time, and associated at the day and person level. Associations at the person level assess the extent to which overall levels of sedentariness or PA during occupational time are correlated with the corresponding levels during leisure time, on average across all individuals (time-independent). Associations at the day level assess the extent to which changes in occupational sedentary or PA behavior, in terms of a specific person-expected average, also implies changes in average leisure-time sedentariness or PA on the same day. Each PA trend is a longitudinal model with a random intercept. Person-level associations are measured by correlations between the two random intercepts (between-effect), day-level associations are measured by within-day residual correlations (within-effect). Day was modelled as categorical, thus without assuming any pre-specified shape of sedentary or PA trend over time. This analysis was done for each type of sedentary or PA separately (sedentary, prolonged sedentary bouts, light-intensity PA and MVPA). The analyses were further stratified by type of work (physical/non-physical). All models were adjusted for age, gender, comorbidity and BMI.

Descriptives of patient characteristics were computed in SPSS version 23. The parallel growth model was estimated with maximum likelihood and robust standard errors in Mplus version 8 [28]. The other statistical analyses were performed in R version 3.5.1 [29].

## Results

We identified n = 256 patients who underwent TKA and were screened for eligibility. Most patients were not eligible as they did not have a paid job or did not work enough hours (< 24 h). N = 57 employees agreed to participate and were enrolled in the study. Of those, n = 51 (89%) wore the accelerometer at least 10 h/day for at least 7 days and were therefore used for analysis. Mean age of the sample was 59 years (SD 4) (range 48–65) and about half were male (47%). A majority of participants were either overweight (33%) or obese (47%) and lived with a partner. Participants worked on average 36 h per week (SD 10), 3 h more than their contract prescribed. Most participants (59%) performed a physical job (Table 1).

**Table 1:** Patient characteristics

Variable	Total sample N = 51	Physical jobs N = 30	Non-physical jobs N = 21
Age [mean (SD) (range)]	59 (4) (48–65)	58 (4) (48–64)	59 (5) (49–65)
Males [n (%)]	24 (47)	15 (50)	9 (43)
Educational level [n (%)]			
Low	13 (26)	12 (40)	1 (5)
Medium	24 (47)	14 (47)	10 (48)
High	13 (26)	4 (13)	9 (43)
BMI [n (%)]			
18.5–24.9	10 (20)	7 (23)	3 (14)
25.0–29.9	17 (33)	10 (33)	7 (33)
≥ 30.0	24 (47)	13 (43)	11 (52)
Comorbidity [n (%)]			
0	9 (18)	5 (17)	4 (19)
1	7 (14)	5 (17)	2 (10)
≥ 2	35 (69)	20 (67)	15 (71)
Living situation [n (%)]			
Alone	7 (14)	1 (3)	3 (14)
With partner	30 (59)	18 (60)	12 (57)
With partner & children	17 (33)	11 (37)	6 (29)
Working hours (mean (SD))			
Actual	36 (10)	34 (8)	32 (6)
Contractual	33 (7)	37 (12)	34 (7)

*BMI* body mass index

### Sedentary Behavior

Table 2 provides descriptive statistics for time in sedentary and prolonged sedentary bouts for the total wearing time during the week and per day, and for occupational and leisure time. During a week participants were sedentary more than half of the time (60.1%). Sedentary behavior showed a stable pattern over working and weekend days. Participants spent 16.5% of their time in prolonged sedentary bouts of at least 30 min.

**Table 2:** Descriptive statistics of time sedentarily and in prolonged sedentary bouts, light-intensity PA and MVPA for total wearing time, occupational time and leisure time, among working TKA patients 1 year after surgery

	All days^a^	Mon	Tue	Wed	Thu	Fri	Sat	Sun
	Mean (SD)	Mean (SD)	Mean (SD)	Mean (SD)	Mean (SD)	Mean (SD)	Mean (SD)	Mean (SD)
Sedentary (% of wearing time)								
Total	60.1 (13.2)	59.5 (12.9)	61.1 (13.8)	60.0 (13.6)	60.8 (11.7)	61.9 (13.9)	54.2 (12.4)	55.2 (14.2)
Leisure	65.2 (13.1)	63.9 (13.6)	65.7 (11.6)	65.8 (14.2)	64.7 (14.0)	66.7 (13.3)	63.0 (12.5)	66.5 (12.5)
Occupational	55.8 (19.5)	56.3 (18.4)	58.1 (20.5)	53.9 (19.5)	56.1 (13.5)	54.1 (22.4)	n.a	n.a
Prolonged sedentary bouts (% of wearing time)								
Total	16.5 (13.1)	16.4 (12.8)	15.7 (12.1)	16.6 (12.1)	16.1 (13.5)	19.0 (15.9)	15.0 (10.3)	14.1 (16.2)
Leisure	20.2 (17.1)	21.1 (18.0)	21.2 (17.3)	19.6 (16.7)	16.7 (16.2)	22.6 (19.9)	19.4 (15.0)	20.5 (16.5)
Occupational	13.1 (16.9)	12.1 (16.0)	12.8 (16.0)	13.1 (14.7)	14.3 (18.5)	13.3 (20.5)	n.a	n.a
Light-intensity PA (% of wearing time)								
Total	36.8 (12.2)	37.0 (12.2)	35.7 (12.9)	36.7 (12.7)	36.3 (10.7)	35.4 (12.4)	43.2 (12.1)	40.8 (13.3)
Leisure	31.4 (11.7)	32.2 (12.5)	30.8 (10.5)	30.9 (12.3)	32.7 (12.3)	30.1 (11.3)	33.6 (11.5)	29.8 (11.5)
Occupational	41.1 (18.5)	40.6 (17.9)	38.5 (18.9)	42.6 (18.3)	40.8 (16.8)	43.7 (21.6)	n.a	n.a
MVPA (% of wearing time)								
Total	3.1 (3.0)	3.5 (2.9)	3.2 (3.2)	3.2 (2.4)	2.9 (3.0)	2.7 (3.3)	2.5 (2.3)	4.0 (4.3)
Leisure	3.4 (4.1)	3.9 (4.1)	3.5 (4.5)	3.3 (4.2)	2.6 (3.4)	3.2 (4.8)	3.4 (3.4)	3.7 (4.2)
Occupational	3.1 (4.0)	3.1 (3.4)	3.4 (5.4)	3.5 (3.8)	3.1 (3.8)	2.2 (4.7)	n.a	n.a
Wearing hours (min/day)^b^								
Total	935.3 (181.4)	960.8 (178.7)	969.3 (193.0)	955.8 (144.8)	975.0 (197.4)	940.4 (197.8)	895.4 (164.0)	850.5 (162.6)
Leisure	618.0 (246.1)	513.7 (187.7)	508.7 (185.1)	514.9 (239.1)	565.8 (240.0)	618.4 (263.8)	820.0 (190.8)	784.2 (185.2)
Occupational	498.0 (148.7)	495.8 (137.0)	499.8 (155.2)	499.7 (124.8)	509.0 (155.8)	483.0 (179.7)	n.a	n.a

*min* minutes, *PA* physical activity, *MVPA* moderate-to-vigorous physical activity

^a^Average across days and within-people

^b^Occupational and leisure wearing time does not always add up because the number of patients at work differed per day

Regarding sedentary behavior during occupational and leisure time, patients were sedentary 65.2% of their leisure time and 55.8% of their occupational time. Sedentary levels during leisure or occupational time were stable over all days. Prolonged sedentary bouts occurred more during leisure time (20.2%) than during occupational time (13.1%).

### Physical Activity Behavior

On average, participants spent 36.8% of the time in light-intensity PA, in a stable pattern over the week. Participants performed on average 3.1% MVPA. Thirty-six (70%) participants met the health-enhancing guideline of at least 150 min MVPA per week. During occupational time, 41.1% of the time was spent in light-intensity PA and 3.1% in MVPA. During leisure time, 31.4% was spent in light-intensity PA and 3.4% in MVPA (Table 2).

### Compensation Effect Between Occupational and Leisure Time

Models for the total sample showed significant within-effects for sedentary (correlation coefficient − 0.56) and light-intensity PA (− 0.41). After stratification for physical and non-physical jobs similar within-effects were found as in the total sample, and additional between-effects for prolonged sedentary bouts and light-intensity PA. Results after stratification into physical and non-physical jobs are reported below and in Table 3. Results of the total sample are presented in the Table 4.

**Table 3:** Compensation effects between occupational and leisure time, stratified by physical and non-physical jobs among working TKA patients, 1 year after surgery

	Physical jobs						Non-physical jobs					
	Occupational time	Leisure time	Between-person effect		Within-person effects		Occupational time	Leisure time	Between-person effect		Within-person effects	
	Mean (SD)	Mean (SD)	Correlation coefficient	p-value	Correlation coefficient	p-value	Mean (SD)	Mean (SD)	Correlation coefficient	p-value	Correlation coefficient	p-value
% sedentary time	24.0 (12.2)	32.1 (12.4)	− 0.29	0.11	− **0.60**	** < 0.001**	36.1 (10.8)	31.0 (6.5)	− 0.29	0.18	− **0.42**	** < 0.001**
% time in sedentary bouts ≥ 30 min	4.2 (6.7)	10.7 (10.2)	0.23	0.42	− **0.31**	**0.03**	10.8 (10.3)	8.5 (6.9)	**0.63**	**0.01**	0.10	0.34
% time in light-intensity PA	25.1 (11.4)	15.7 (9.7)	− **0.40**	**0.01**	− **0.35**	** < 0.001**	15.0 (8.0)	14.7 (6.6)	0.26	0.51	− **0.53**	** < 0.001**
% time in MVPA	1.6 (2.2)	1.5 (2.2)	n.a.^a^		n.a.^a^		1.4 (1.2)	1.7 (2.1)	n.a.^a^		n.a.^a^	

Bold values indicate statistical significance p < 0.05

^a^Non-convergent models and skewed variable

Among participants with physical jobs, a significant between-person effect (correlation coefficient − 0.40) of light-intensity PA was observed between occupational and leisure time. This implies that participants with physical jobs compensated for their occupational light-intensity PA routinely with less light-intensity PA during leisure time. A significant within-person effect was also observed for sedentary time (correlation coefficient − 0.60), prolonged sedentary bouts (− 0.31) and light-intensity PA (− 0.35). This means that an increase of sedentary time, time in prolonged sedentary bouts and light-intensity PA at work on a specific day were negatively associated with these leisure-time PA levels the same day, i.e. less sedentary behavior or light-intensity PA (Table 3).

Among participants with non-physical jobs, a significant between-person effect (correlation coefficient 0.63) was observed for prolonged sedentary bouts between work and leisure time. This coefficient indicates that participants who had more prolonged sedentary bouts at work also had longer prolonged sedentary bouts during leisure time in a week. Further, a significant within-person effect was observed for sedentary behavior (correlation coefficient − 0.42) and light-intensity PA (− 0.53), which implies that on specific days when participants had more sedentary behavior or light-intensity PA at work, this was compensated by less sedentary or light-intensity PA during leisure time (Table 3).

MVPA models were non-convergent due to skewed distributions so they could not be analyzed. All models were adjusted for age, gender, comorbidity and BMI. Adjusted models showed similar results, therefore unadjusted models were tabulated and reported.

## Discussion

The results of the present study showed that working-age TKA patients spend most of their daily time sedentarily. Approximately a quarter of their sedentary time was spent in prolonged sedentary bouts of 30 min or more. Sedentary behavior occurred more during leisure time than during occupational time. PA behavior was mainly light-intensity, while MVPA levels were low during the day. Thirty percent of the patients did not fulfill the PA guideline of at least 150 min MVPA per week. Patients performed more light-intensity PA during occupational time than during leisure time, and MVPA levels were equally distributed. On a routine basis, those with physical jobs compensated for their occupational light-intensity PA with less light-intensity PA during leisure time. Participants in non-physical jobs did not compensate for their occupational prolonged sedentary time, while they routinely continued their prolonged sedentary bouts during leisure time.

Results from the present study could only be compared with accelerometer data from TKA patients of all ages and with a general population, and results were generally similar. The observed sedentary levels (60%), prolonged sedentary bouts (16%) and light-intensity PA levels (37%) of the present study were also reported among TKA patients of all ages—64%, 13% and 35%, respectively [30, 31]. A general population derived from pooled data of four European countries also showed 61% sedentary time, 15% prolonged sedentary bouts and 35% light-intensity PA [32]. Patients were sedentary most of the time, and only a quarter of the sedentary time was accrued in prolonged sedentary bouts. This implies that our participants were likely standing up during work and leisure time on a regular basis.

Participants performed low amounts of MVPA, but 70% reached the updated health-enhancing PA guideline of at least 150 min MVPA per week. Similar percentages have been found in a general European population [32]. However, this updated guideline has not been used among TKA patients yet. Thirty percent did not meet the guideline, which puts them at unnecessary risk for chronic diseases. We used the updated PA guideline for Americans [8], while previous studies used the former guideline of minimum 30 min MVPA 5–7 days/week in bouts of at least 10 min. Two studies that examined compliance with the former guideline by means of accelerometer measurements showed that between zero and 5% of TKA patients of all ages met this norm [33, 34]. In a post-hoc analysis we examined how many participants met the former PA guideline; this revealed that only 2 participants (4%) met that norm, in line with previous observations. It is therefore important to note that using these PA guidelines may lead to major discrepancies in interpretation.

By analyzing the association between PA levels during occupational and leisure time, stratified by physical and non-physical jobs, we found several within-day effects. It is reasonable to expect that in specific days with higher intensities of occupational PA this will be naturally compensated for with less PA during leisure time on the same day, regardless of the individual’s health. The routine compensation effects in the present study are of special interest. We found that participants with physical jobs may compensate on a routine basis by performing less light-intensity PA during leisure time, and participants in non-physical jobs may not compensate at all while continuing to spend time in prolonged sedentary bouts during leisure time. The compensation effect of occupational light-intensity PA has been found earlier in a general working population [21], but not specifically for physical or non-physical jobs yet. For physical jobs the compensation effect of less light-intensity PA during leisure time may depend on the demands made of participants during occupational time. Working-age TKA patients are known to have problems with getting to and from work and around the workplace, pace of work and meeting job demands [35], which might explain the compensation style during leisure time. In addition, the lack of compensation for occupational prolonged sedentary bouts among those with a non-physical job is worrisome and contrasts with two studies in which TKA patients reported problems with sitting for long periods [35, 36]. These data are in line with a large epidemiological study using self-report measures, where individuals sitting for long periods at work also sat a lot during their leisure time [37]. Finally, compensation might also exist between working days and a day off (either on the weekend or on a weekday). In a sensitivity analysis we therefore analyzed whether compensation effects exist in PA levels on the day off after at least three consecutive working days. We chose three consecutive working days as we included many part-time participants and assumed that they will compensate on their first day off. This revealed no statistically significant or clinically relevant results.

### Strengths and Limitations

Several strengths and limitations of our study should be mentioned. A strength is that, to our knowledge, it is the first study to examine PA levels among working-age TKA recipients and make a distinction between occupational time and leisure time to gain further insight into the PA behavior of working patients. Moreover, PA was assessed by accelerometers, which are more reliable and valid than self-reported measures that are subject to recall and social desirability bias. Finally, the longitudinal study design, analyzed using a multilevel model, could separate the within-effects and between-effects, providing additional insight into TKA patients’ compensation effects.

A study limitation was the small sample size, which limited analysis of MVPA performed by a few participants and showed a skewed distribution. The present study does give a first glimpse into PA behavior of working TKA patients and stresses the need for further accelerometer measurements in future studies. Secondly, ours was a widely used cut-off point of < 100 cpm for sedentary behavior, but cut-off points or activity intensity thresholds vary in the literature and there is no universally accepted cut-off point [38]. The same applies to selected epoch lengths and criteria for determining a sedentary bout. In addition, commuting hours were allocated to leisure time, which might have led to a small increase in physical activity during leisure time. As the majority of the study sample were sedentary and performed mostly light-intensity physical activity, future studies might focus on the details within these categories. New accelerometry intervals might be useful for these future studies [38]. Lastly, the accelerometers did not record any swimming activities as they were not waterproof. PA levels might therefore be slightly underestimated, but patients’ logs revealed that only one participant swam every morning for 15 min. We did not assess any pain or function levels among the participants, which limited the interpretability of the PA levels.

### Implications and Recommendations

This study showed that the 70% of patients who met the guideline can gain additional benefits with additional PA, but the 30% of patients who did not fulfill the health-enhancing PA guideline should be primarily stimulated to become more active. A higher level of PA is associated with health-enhancing benefits such as better bone quality, improved coordination and more muscle strength, which are especially important for people who have undergone TKA [9]. Such benefits transcend known reductions of risks for diabetes mellitus or cardiovascular disease, or all-cause mortality. Sitting behavior should preferably be changed to MVPA. Given the low amount of MVPA currently performed in the present study, performing light-intensity PA is a more reasonable expectation. A meta-analysis also showed that decreasing sitting time and a change to any PA intensity is already associated with substantially reduced risks of premature mortality [8, 39, 40].

The present study showed that non-physical jobs are more exposed to uninterrupted periods of sitting than physical jobs, suggesting the need to encourage PA among non-physical workers. When trying to activate occupational PA it is important to note that interventions at the workplace may not necessarily lead to an increase of total PA behavior, as individuals compensate PA behavior at work with less PA during leisure time. Organizational encouragement of PA, for example by using break-reminder software, retrieving printer output, getting coffee from the other end of the hall or taking lunchtime time walks might nonetheless be a successful first step toward motivating and activating TKA patients.

## Conclusion

Working-age TKA patients spend a majority of their time behaving sedentarily 1-year after TKA surgery. A majority met the international guideline of health-enhancing PA (> 150 min MVPA per week). Participants in physical jobs compensated for their occupational light-intensity PA by less light-intensity PA during leisure time. Participants in non-physical jobs continued their occupational prolonged sedentary bouts during leisure time, which suggests that they do not compensate for their prolonged sedentary time at work. These findings stress the need to encourage PA among TKA patients who do not meet the PA guidelines and among those with non-physical jobs.

## Appendix

See Table 4.

**Table 4:** Associations of work and leisure time sedentarily and in sedentary bouts, light-intensity PA and MVPA for five consecutive working days (Monday–Friday)

	Occupational time	Leisure time	Between-persons effect	P-value	Within-person effect	P-value
	Mean (SD)	Mean (SD)	Correlation coefficient		Correlation coefficient	
Total wearing time (min/day)	498.0 (148.7)	476.8 (173.6)				
% sedentary time	29.0 (13.1)	31.6 (10.4)	− 0.27	0.06	** − 0.56**	** < 0.001**
% time in sedentary bouts ≥ 30 min	6.9 (9.0)	9.8 (9.1)	0.22	0.27	− 0.14	0.22
% time in light-intensity PA	21.0 (11.3)	15.3 (8.5)	− 0.18	0.13	** − 0.41**	** < 0.001**
% time in MVPA	1.5 (1.9)	1.6 (2.1)	0.19	0.43	− 0.01	0.93

Bold values indicate statistical significance p < 0.05

*min* minutes
